# Deep Learning Models for Fast Retrieval and Extraction of French Speech Vocabulary Applications

**DOI:** 10.1155/2022/4286659

**Published:** 2022-07-08

**Authors:** Man Xu

**Affiliations:** School of Zhejiang International Studies University, Hangzhou 310023, China

## Abstract

Due to the large French vocabulary, how quickly retrieve and accurately identify the required vocabulary is still a big challenge in French learning. In view of the above problems, we introduce a deep learning algorithm in this study to upgrade and optimize the retrieval system of French words and optimize the acquisition speed of speech words data and the recognition accuracy of speech words, so as to meet the needs of users for word retrieval. The results show that the two training methods of SGD synchronous update network and alternate update network parameters for fast retrieval and extraction of French speech vocabulary reduce from a maximum of 11.65% to 4.25% in the WER criterion, with a maximum reduction of 7.4%; the two training methods of SGD synchronous update network and alternate update network parameters for fast retrieval and extraction of French speech vocabulary reduce from a maximum of 13.52% to 4.4% in the SER criterion. The training methods of fast retrieval and extraction of the SGD synchronous update network and alternate update network parameters in French speech vocabulary reduced from the highest 582 ms to 351 ms in the response time criterion, with a maximum reduction of 8.84%; the maximum reduction of 39.7%. In French speech vocabulary, SGD synchronous updating network and alternating updating network parameter algorithm are used to quickly retrieve and extract French words. When the number of iterations reaches 120, the model fitting accuracy of the training set reaches 90.05%, while the model can reach 94.5% in the test set. The system has a stronger generalization ability and a higher speech vocabulary recognition rate to meet the practical requirements.

## 1. Introduction

The new era has new requirements for French-language education and a mission to cultivate high-quality and international talents for national strategic development [1, 2]. As a compulsory course in all universities, the teaching of French should keep pace with the times and lay the foundation for cultivating more high-quality talents. With the popularity and use of the Internet, a large number of users have joined the network, and according to the statistics, as of June 2018, the number of Chinese Internet users was 802 million. The huge number of netizens brings a large amount of data. According to IDS estimates, the scale of the global data circle will continue to expand in the next few years. By 2025, the world will create and replicate 163 ZB of data [3, 4]. With the proliferation of text data, it is more and more difficult for users to obtain target data from text databases. Similarly, it is also faced with huge challenges in the fast retrieval and recognition of French vocabulary. A student who speaks more than one language multiplies his or her chances in the national and international job markets. On five continents, 200 million people speak French. French is a major language used for international communication and is one of the most studied foreign languages after English, ranking ninth among the most spoken languages in the world [5, 6]. Mastering French allows one to communicate with French speakers from all continents while expressing another perspective on the world, and to benefit from well-informed international media in French (e.g., TV5, France 24, and Radio France International) [7, 8]. However, French vocabulary is the most crucial basic part in the process of learning French, and if it is difficult to master even the vocabulary, then let alone master the language; at the same time, due to the large vocabulary base of French, it is still a major challenge in learning French to quickly retrieve and precisely identify the required words [4, 9, 10].

French is an analytical language that has a structure of thought and promotes a critical spirit. French is the language used by great philosophers (such as Descartes, Sartre, and Derrida) and famous scientists (such as the Curies, Pasteur, and Georges Charpak). Students are learning French while learning to make inferences and present different points of view, and learning such methods can be very useful in later discussions or negotiations [11–13]. At present, the first step in learning French is to search for relevant vocabulary, and information retrieval techniques originated from the search and indexing of library materials. The original retrieval system had a single function and could only perform simple searches. Nowadays, it is possible to retrieve information from the whole network, and a lot of new models and methods have been applied to the retrieval system. Aoxiao [14] et al. developed an image retrieval system based on a new deep metric learning algorithm and found that their image retrieval model based on deep metric learning is highly efficient in CXR retrieval, diagnosis, and prognosis with high practical significance through experimental comparison. Da et al. [15] proposed a deep learning-based dual encoder retrieval (DER) model. Pang [16] et al. proposed a new method that uses deep learning techniques to extract high-level and compact features from biomedical images. The deep feature extraction process utilizes multiple hidden layers to capture a large number of feature structures of high-resolution images and represent them at different levels of abstraction, thus improving the indexing and retrieval performance of biomedical images. Beltrán [17] et al. used the VQA model for deep multimodal learning to combine visual and textual representations. Hao [18] investigated the problems related to relevance matching between queries and documents. Gozuacik et al. [19] utilized deep neural networks and natural language processing methods. The above literature analysis shows that deep learning is powerful with superior results in computer vision, image retrieval, speech retrieval, and biological image correlation.

Vocabulary information retrieval refers to the retrieval of vocabulary information from databases to meet user needs and query-related content. The ultimate effect of vocabulary retrieval recognition is to pave the way for French language learning. However, nowadays, vocabulary retrieval has the problems of low retrieval accuracy, slow retrieval speed, and low recognition rate. Therefore, in this study, we introduce the deep learning algorithm to upgrade and optimize the French vocabulary retrieval system, from the speed of acquiring vocabulary data to the accuracy of vocabulary recognition, so as to meet the user's needs for vocabulary retrieval.

## 2. Concepts Related to Phonetic Word Search

**Figure 1:**
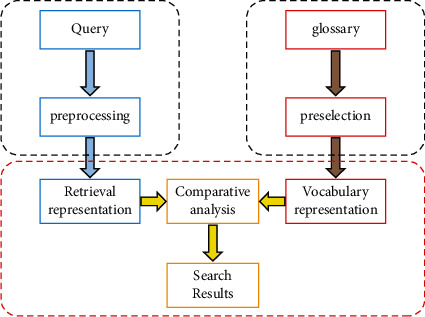
Vocabulary search framework.

Vocabulary is the most critical and fundamental part of learning French, and mastering it is the first step to success. Vocabulary retrieval and recognition are key techniques in learning vocabulary. The basic task of vocabulary retrieval is to find the answer to the user's given needs from a large database of words. The retrieval query is a literal description of the user's retrieval needs, and the vocabulary is the basic object of retrieval and the basic object returned. To further enhance the effectiveness of the algorithm, the user's behavior on the final feedback results will be used as feedback on the retrieval results, thus further enhancing the retrieval effect [20–22]. The overall framework of vocabulary retrieval and recognition is shown in Figure 1, where each node corresponds to each module of information retrieval.

In the vocabulary retrieval system, the retrieval model and index are the two most critical parts. The retrieval system generally consists of three parts: processing module, retrieval module, and user interface module. The retrieval module can be subdivided into vocabulary index module, user query module, and sorting module. The vocabulary index module first preprocesses all the indexed documents and then performs the inverted index [22–24]; the user query module first preprocesses the user query obtained from the previous interface and quickly retrieves the relevant vocabulary from the inverted index, and then the lexical matching model is used to finally draw a matching conclusion. In order to improve the retrieval accuracy, before the final word feedback, the retrieval system will use the user's result to make further corrections to improve retrieval accuracy [25, 26].

## 3. Theoretical Foundation of Speech Recognition Based on Deep Learning

In the field of speech signal processing, deep neural networks were first successful in the classification task of speech recognition. After that, many scholars started to apply deep neural networks to speech enhancement tasks. In addition, MMSE objective function-based speech enhancement minimizes the mean square error between the estimated value of the network output and the labeled target value during training, and it treats each time-frequency point as equally important, taking into account the distribution characteristics of the speech spectrum; the trained neural network gets an average optimal result at the time of enhancement, which solves the serious problem of oversmoothing and speech distortion and loss under low signal-to-noise ratio.

### 3.1. Deep Neural Network (DNN) Architecture

**Figure 2:**
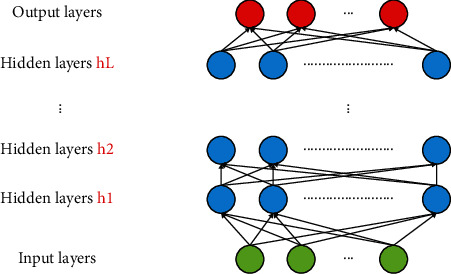
Deep neural network model structure.

DNN is a deep network structure based on a shallow artificial neural network with stronger nonlinear expression capability by adding hidden layers [27–29]. The network is composed of an input layer, an output layer, and an intermediate *L* hidden layer, which is characterized by the fact that individual neurons within the same layer are not connected to each other and the neurons in adjacent layers are fully connected to each other, as shown in Figure 2.

Suppose the number of neurons in the *l*th layer is *n*_*l*_, the input vector is *z*^*l*^, the output vector is *h*^*l*^, and given a training sample *x* with *h*^0^ = *z*^0^ = *x*, then

(1) $$\begin{array}{c} z^{l}=W^{l}z^{l-1}+b^{l}, \end{array}$$

where *W*^*l*^ is the weight matrix from the (*l*-1)th layer to the lth layer and *b*^*l*^ represents the bias matrix of the *l*th layer. Then, there are

(2) $$\begin{array}{c} h^{l}=f_{1}\left(z^{l}\right), \end{array}$$

where *f*_1_ represents the activation function of the *l*th layer and the common form is ReLU, tanh, sigmoid, and softmax.

Speech recognition is a multiclassification problem, so the softmax function is chosen for the activation function of the output layer, and the final output of the DNN is assumed to be *y* = *h*^*L*+1^, which takes the following form:

(3) $$\begin{array}{c} y=\text{softmax}\left(z^{l}\right)\\ =\frac{\exp\left(z^{L}\right)}{\sum_{k=1}^{n^{L}}\text{exp}\left(z^{Lk}\right)}, \end{array}$$

where *z*^*Lk*^ denotes the *k*th component of the vector *z*^*L*^.

### 3.2. Derivation of New Objective Function

The DNN inputs the LPS features of the *D*-dimensional noisy speech signal (2*τ*+1), and by extending the frames of the input features, the prediction error is defined as follows:

(4) $$\begin{array}{c} e_{n}=x_{n}-{\hat{x}}_{n}\left(y_{n-r}^{n+r},W\right), \end{array}$$

where *y*_*n*−*r*_^*n*+*r*^ is the *D*-dimensional LPS feature of each extended *τ* frame on the left and right of the input feature, *x*_*n*_ is the learning target of the network, and *W* is the neural network parameter.

Assuming that the prediction errors in each dimension are independently distributed and using GGD to model the prediction errors in each dimension, the *e*_*n*_ distribution function is as follows:

(5) $$\begin{array}{c} y\left(e_{n}\right)=\prod_{d=1}^{D}p_{GGD}\left(e_{n},d\right), \end{array}$$

where *e*_*n*_ represents the prediction error in dimension.

Assuming that the prediction errors in each dimension obey the same shape distribution, that is, *β* has a fixed value for all dimensions, then the expression is as follows:

(6) $$\begin{array}{c} p\left(x_{n},W,\alpha \right)=\prod_{d=1}^{D}p_{GGD}\left(x_{n,d}-{\hat{x}}_{n,d}\left(y_{n-r}^{n+r},W\right),\alpha_{n},\beta \right), \end{array}$$

where *α* represents the scale parameter of the prediction error distribution GGD on the *d*th dimension.

Given *N* parallel speech data, assuming that the conditional probability distribution is obtained by independent sampling and taking the logarithm of both sides, then the following formula can be obtained:

(7) $$\begin{array}{c} \ln\;p\left(X,W,\alpha \right)=\sum_{n=1}^{N}\sum_{d=1}^{D}\left(\ln\frac{\beta }{2\alpha_{d}\Gamma \left(1/\beta \right)}-\frac{{\left|x_{n,d}-{\hat{x}}_{n,d}\left(y_{n-r}^{n+r},W\right)\right|}^{\beta }}{\alpha_{d}^{3}}\right), \end{array}$$

where *lnβ*/2*α*_*d*_(1/*β*) is a constant.

In addition, the method of maximum likelihood estimation is introduced in this study to optimize *W* and *α* at the same time. Maximizing the log-likelihood function is equivalent to minimizing the formula as follows:

(8) $$\begin{array}{c} E\left(W,\alpha \right)=N\sum_{d=1}^{D}\ln\alpha_{d}+\sum_{n=1}^{N}\sum_{d=1}^{D}\left(\frac{{\left|x_{n,d}-{\hat{x}}_{n,d}\left(y_{n-r}^{n+r},W\right)\right|}^{\beta }}{\alpha_{d}^{\beta }}\right). \end{array}$$

Assuming that the prediction errors in each dimension obey the equal variance distribution, that is, the GGD scale parameters in each dimension are the same, then the following formula can be obtained:

(9) $$\begin{array}{c} E\left(W\right)={\sum_{n=1}^{N}\sum_{d=1}^{D}\left|x_{n,d}-{\hat{x}}_{n,d}\left(y_{n-r}^{n+r},W\right)\right|}^{\beta }, \end{array}$$

where, when *β* is 1, the above formula is the minimum mean absolute error, and when *β* is 2, the above formula is the minimum mean square error.

### 3.3. Training Algorithms

The network parameters are generally updated in a minimum batch mode using the stochastic gradient descent method during network training. The new objective function proposed in this section, that is, the objective function in the small-batch sample update mode is obtained as follows:

(10) $$\begin{array}{c} E\left(W,\alpha \right)=M\sum_{d=1}^{D}\ln\alpha_{d}+\sum_{n=1}^{N}\sum_{d=1}^{D}\left(\frac{{\left|x_{m,d}-{\hat{x}}_{m,d}\left(y_{m-r}^{m+r},W\right)\right|}^{\beta }}{\alpha_{d}^{\beta }}\right), \end{array}$$

where *m* is the minimum batch sample size.

This section proposes two training algorithms: one is to use SGD to update network parameters *W* and *α* synchronously; the other is to update network parameters *W* and *α* alternately. The detailed introduction is as follows:

(1) The parameter *α* of the network parameter gray-sum distribution function is synchronized by the error back propagation algorithm of SGD, and the updated formula is as follows:
  (11) $$\begin{array}{c} W^{\left(t+1\right)}=W^{\left(t\right)}-\eta \frac{\nabla E_{W}}{M},\\ \alpha^{\left(t+1\right)}=\alpha^{\left(t\right)}-\zeta \frac{\nabla E_{\alpha }}{M}, \end{array}$$
where *t* represents the number of iterations, *η* and *ξ* represent the learning rate of network parameters *W* and *α*, and ∇*E*_*W*_ and ∇*E*_*α*_ represent the partial derivative of the objective function with respect to *W* and *α*. In addition, the calculation formula of ∇*E*_*α*_ is as follows:
  (12) $$\begin{array}{c} \nabla E_{\alpha }=\frac{M}{\alpha_{d}}-\frac{\beta }{{\left(\alpha_{d}\right)}^{\beta +1}}\sum_{m=1}^{M}{\left|x_{m,d}-{\hat{x}}_{m,d}\left(y_{m-r}^{m+r},W\right)\right|}^{\beta }, \end{array}$$
where ${\hat{x}}_{m,d}$ is an abbreviation for ${\hat{x}}_{m,d}\left(y_{m-r}^{m+r},W\right)$.
(2) Another training algorithm is to alternately update the network parameter W and the distribution function parameter *α.* When the network parameter *W* is fixed, the closed-form solution of the distribution function parameter *α* can be obtained as follows:
  (13) $$\begin{array}{c} \alpha_{d}=\frac{\beta }{M}\sum_{m=1}^{M}{\left|x_{m,d}-{\hat{x}}_{m,d}\left(y_{m-r}^{m+r},W\right)\right|}^{\beta }. \end{array}$$

In algorithm (2), *α* is updated by using the closed solution obtained under the maximum likelihood criterion, which saves the trouble of manually adjusting the learning rate *ξ* in algorithm (1) when using SGD's reverse error propagation algorithm to update, and the algorithm is more robust.

### 3.4. Speech Lexical Signal Preprocessing

Speech vocabulary signal preprocessing is the basic processing of speech analog signal before feature extraction, and its purpose is to eliminate the impact on the quality of speech signal due to the human articulation organ itself and the equipment used to collect speech signal, such as mixing and high harmonic distortion. It tries to ensure a more uniform and smooth signal for the subsequent speech vocabulary processing and provide a high-quality signal for feature extraction. Preprocessing technology is the premise and foundation of speech recognition, and its key technologies mainly include pre-emphasis, frame-splitting plus windowing, and endpoint detection.

The power spectrum of the voice signal will fall in the frequency range of 6 d/B times in the high-frequency band. Before the voice signal processing, the high-frequency band needs to be increased to make the power spectrum flatter, which is beneficial to the analysis of spectrum or channel parameters. Pre-emphasis technology can remove effects such as lip radiation by boosting the high-frequency band of the speech signal. Pre-emphasis is usually implemented with a first-order high-pass digital filter, and the formula for its transfer function is as follows:

(14) $$\begin{array}{c} H\left(z\right)=1-\mu Z^{-1},0.9\leq \mu \leq 1.0, \end{array}$$

where *μ* represents the pre-emphasis coefficient, generally 0.94. In the time domain, assuming the input original signal is S(*n*), the pre-emphasized speech signal is as follows:

(15) $$\begin{array}{c} S^{\prime }\left(n\right)=S\left(n\right)-\mu S\left(n-1\right). \end{array}$$

After the speech signal is preprocessed, it needs to be framed and windowed, which is beneficial to the subsequent operations such as feature extraction of the speech signal. The specific formula is as follows:

(16) $$\begin{array}{c} s_{w}=\sum_{-\infty }^{+\infty }T\left[s\left(n\right)\right]w\left(n-m\right), \end{array}$$

where *T* is a certain linear or nonlinear operation, *s*(*n*) is the speech signal before windowing, *w* (*n*-*m*) is a certain window function, and *s*_*w*_ (*n*) is the speech signal after windowing.

## 4. Experimental Verification and Comparative Analysis

### 4.1. Comparative Analysis of Accuracy of Speech Word Recognition Results

This study adopts word error rate (WER) and sentence error rate (SER) as the main evaluation criteria for speech recognition. WER represents the relationship between the recognized word sequence and the standard word sequence. The continuous speech recognition results are generally expressed in the form of word sequences. The dynamic programming algorithm is used to align the recognition results with the correct label series and then compare them. There are three types of errors: insertion error *I*, deletion error *D*, and substitution error *R*. Assuming that the total number of word sequences is *N*, the definition of WER is as follows:

(17) $$\begin{array}{c} WER=\frac{I+D+R}{N}\times 100\%. \end{array}$$

SER represents the probability of recognizing a correct sentence. Whenever a sentence has a word error, the sentence is considered to be incorrectly recognized, and SER is the number of incorrectly recognized sentences *m* over the total number of sentences *M*, which is defined as follows:

(18) $$\begin{array}{c} SER=\frac{m}{M}\times 100\%. \end{array}$$

**Figure 3:**
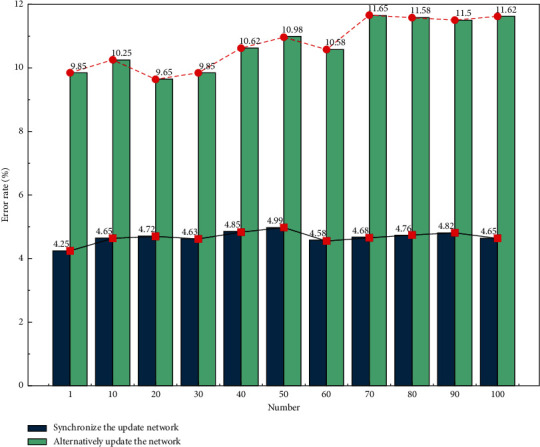
WER analysis graph of two training methods of SGD synchronous update network and alternate update network parameters.

It is worth mentioning that Figure 3 shows the WER analysis for both training methods of SGD synchronous update network and alternate update network parameters. It can be seen that by using alternate update network parameters, it is able to reduce the WER from a maximum of 11.65% to 4.25%, with a maximum reduction of 7.4%. The reason for this is that when increasing N causes more neurons to be inactivated, the sparsity of the entire neural network is increased. It can also be found that as the sparsity increases, the alternate update network training method also has a certain degree of improvement in recognition correctness and computational efficiency with better recognition and lower computational effort.

**Figure 4:**
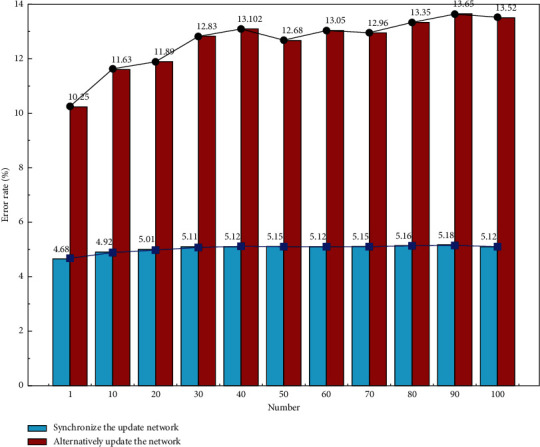
Comparison graph of SER analysis between two training methods of SGD synchronous update network and alternate update network parameters.

It is worth mentioning that Figure 4 shows the SER analysis of the two training methods of SGD synchronous update network and alternate update network parameters. It can be seen that by using the alternate update network parameter training method, the SER can be reduced from a maximum of 13.52% to 4.68%, with a maximum reduction of 8.84%. This indicates that the speech bottleneck features extracted based on the alternating update network parameter training method can improve the speech recognition efficiency to a certain extent. The reason for this is that using the sparse regular term as the penalty term of the target function improves the generalization ability of the target function to a certain extent, thus increasing the recognition rate accordingly.

**Figure 5:**
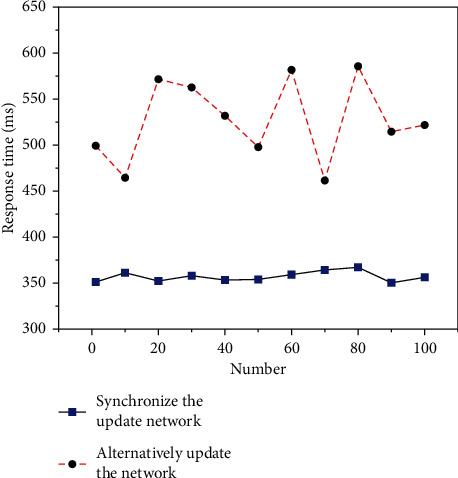
Comparison chart of the response time analysis between two training methods of SGD synchronous update network and alternate update network parameters.

It is worth mentioning that Figure 5 shows the time analysis of the two training methods of SGD synchronous update network and alternate update network parameters. It can be seen that by using the alternate update network parameter training method, the time consumption can be reduced from a maximum of 582 ms to 351 ms, with a maximum reduction of 39.7%. This indicates that the extracted speech based on the alternating update network parameter training method can provide timely feedback to predict the final result and provide faster speech recognition information for the subsequent speech recognition process.

**Figure 6:**
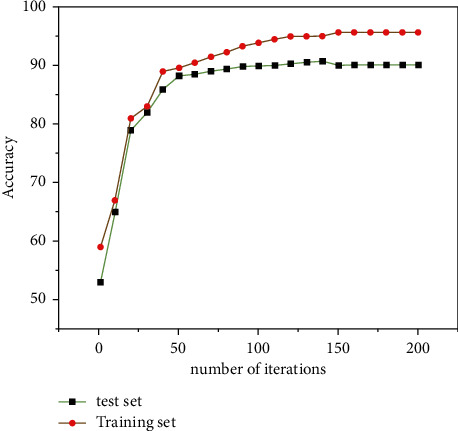
Iteration results of CNN-GRU model training set and test set.

It is worth mentioning that Figure 6 shows the graph of the iteration results of the training set and the test set of the CNN-GRU model. It can be seen from Figure 6 that the network results gradually regionally converge as the number of iterations increases, and when the number of iterations reaches 120, the accuracy of the training set satisfies at 90.05%, while the accuracy of the test set satisfies at 94.5%. This indicates that the CNN-GRU network model is able to retrieve French speech words quickly.

## 5. Conclusion

In this study, we provide a basic description of the concept of multicommunication framework and neural network algorithm and introduce the DNN algorithm structure and computational flow for French speech retrieval recognition. We also compare the advantages and disadvantages of two training methods, SGD synchronous update network, and alternate update network parameters using three criteria: WER, SER, and response time. The optimization of fast retrieval of French speech vocabulary by deep learning methods improves the system in terms of the accuracy of retrieval of individual words of speech, the accuracy of sentence retrieval, and the response time of retrieval, and comparing the accuracy of the model under a different number of iterations, the conclusions meet the practical needs of retrieval. We hope that our proposed deep learning retrieval optimization can add to the French speech vocabulary learners. The specific results are as follows:

1. Vocabulary information retrieval is the retrieval of vocabulary information from a database to meet user needs and query-related content. The ultimate effect of lexical retrieval recognition is to pave the way for French language learning. In our proposed model, fast retrieval and extraction of French phonetic words are reduced from a maximum of 11.65% to 4.25% in the WER rubric, with a maximum reduction of 7.4%.
2. As complete and correct recognition is crucial in the application, it is worth mentioning that our training method based on alternating update network parameters for fast retrieval and extraction of French speech words reduces from a maximum of 13.52% to 4.68% in the SER rubric, with a maximum reduction of 8.84%.
3. In addition, in the fast retrieval system, the response time for retrieval is a key factor affecting the user experience. In our model, the response time criterion is reduced from a maximum of 582 ms to 351 ms, with a maximum reduction of 39.7%.
4. In French speech vocabulary, SGD synchronous updating network and alternating updating network parameter algorithm are used to quickly retrieve and extract French words. When the number of iterations reaches 120, the model fitting accuracy of the training set reaches 90.05%, while the model can reach 94.5% in the test set.

## Data Availability

The dataset can be obtained from the author upon request.
